# Massive Intraventricular Thrombosis in a Young Woman with Idiopathic Dilated Cardiomyopathy

**DOI:** 10.5935/abc.20150131

**Published:** 2015-12

**Authors:** Natalia Lorenzo, Jorge A. Restrepo, Maria Cruz Aguilera, Daniel Rodriguez, Rio Aguilar

**Affiliations:** 1 Hospital Universitario Infanta Cristina, Madri - Espanha; 2 Hospital Universitario De La Princesa, Madri - Espanha

**Keywords:** Intraventricular Thrombosis, Cardiogenic Shock, Ventricular Dysfunction

A 36-year-old woman, with no remarkable medical or family history, was admitted to the hospital in cardiogenic shock. Transthoracic echocardiography (TTE) revealed severe biventricular dilation and dysfunction. Several mobile masses consistent with thrombi were attached to the apex and protruding into the left ventricle (LV) beyond the mid-ventricular level (Figure 1A-D. Multiplane-view (A). 2-Chamber-view with (B) and without (C) Sonovue®. Short-axis-view (D)). Internal hypoechogenic regions suggestive of colliquative tissue secondary to clot lysis were identified (arrow). Coronary angiogram was normal. Cardiac magnetic resonance imaging did not provide further information. Inotropic drugs and unfractionated heparin were started. Systemic fibrinolysis was discarded because of high risk of thrombus fragmentation. The patient was also rejected for surgery considering high peri-operative risk due to cardiogenic shock. Five days after admission, massive stroke in the left middle cerebral artery occurred (Figure 1E[*]). It was established in 30 minutes before any reperfusion strategy was possible. The patient was dismissed for further treatment and died one week later.

**Figure:**
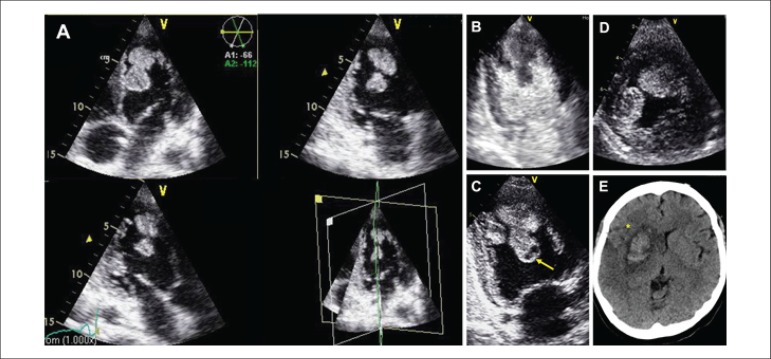


Any condition with severe LV systolic dysfunction increases the probability of intraventricular thrombus formation. Incidence of systemic embolization is low; nevertheless, it increases in cases of large, protuberant and highly mobile thrombi. Therapeutic approach in this scenario is controversial. It is generally agreed that anticoagulation should be the initial therapy in most of cases, but there are no specific recommendations regarding thrombolysis or thrombectomy.

TTE is the gold standard technique for diagnosis and stratification of embolic risk, since it allows accurate assessment of morphology, mobility and point of attachment of the clot.

Conception and design of the research and Analysis and interpretation of the data: Lorenzo N; Acquisition of data: Lorenzo N, Restrepo JA, Aguilera MC; Writing of the manuscript: Lorenzo N, Rodriguez D; Critical revision of the manuscript for intellectual content: Lorenzo N, Aguilar R.

